# Mortality by road traffic injuries in Brazil (2000–2016): capital cities versus non-capital cities

**DOI:** 10.11606/s1518-8787.2020054001703

**Published:** 2020-11-17

**Authors:** Érika Carvalho de Aquino, José Leopoldo Ferreira Antunes, Otaliba Libânio de Morais

**Affiliations:** I Instituto de Patologia Tropical Programa de Pós-Graduação GoiâniaGO Brasil Universidade Federal de Goiás. Instituto de Patologia Tropical e Saúde Pública. Programa de Pós-Graduação em Medicina Tropical e Saúde Pública. Goiânia, GO, Brasil; II Faculdade de Saúde Pública Departamento de Epidemiologia São PauloSP Brasil Universidade de São Paulo. Faculdade de Saúde Pública. Departamento de Epidemiologia. São Paulo, SP, Brasil; III Instituto de Patologia Tropical e Saúde Pública Departamento de Epidemiologia GoiâniaGO Brasil Universidade Federal de Goiás. Instituto de Patologia Tropical e Saúde Pública. Departamento de Epidemiologia. Goiânia, GO, Brasil

**Keywords:** Accidents, Traffic, mortality, Spatial Analysis, Time Series Studies

## Abstract

**OBJECTIVES:**

To compare the magnitude and trend of mortality by road traffic injuries (RTI) in the capitals and other municipalities of each Brazilian state between 2000 and 2016.

**METHODS:**

A time series analysis of mortality rates by RTI standardized by age was performed, comparing the capitals and the cluster of non-capital municipalities in each state. Data on deaths were obtained from the *Sistema de Informações sobre Mortalidade* (SIM – Mortality Information System). RTI deaths were considered to be those, whose root cause was designated by ICD-10 codes V01 to V89, with redistribution of garbage codes. To estimate mortality rates, we used the population projections of the Brazilian Institute of Geography and Statistics (IBGE) from 2000 to 2015 and the population estimated by polynomial interpolation for 2016. The trend analysis was performed using the Prais-Winsten method, using the Stata 14.0 program.

**RESULTS:**

There were 601,760 deaths due to RTI in the period (114,483 of residents in capital cities). Mortality by RTI did not present an increasing trend in any of the Capitals in the period under study. Among non-capital municipalities, the trend was growing in 14 states. The greatest increase was observed in Piaui (AIR = 7.50%; 95%CI 5.50 – 9.60). There was a decreasing trend in RTI mortality in 14 capitals, among which Curitiba showed the greatest decrease (AIR = −4.82%; 95%CI −6.61 – −2.92). Only São Paulo and Rio Grande do Sul showed a decreasing trend in mortality by RTI in non-capital cities (AIR = 2.32%; 95%CI −3.32 – −1.3 and AIR = 1.2%, 95%CI −2.41 – 0.00, respectively).

**CONCLUSIONS:**

We conclude that RTI mortality rates in non-capital cities in Brazil showed alarming trends when compared with those observed in capital cities. The development of effective traffic safety actions is almost always limited to Brazilian capitals and large cities. Municipalities with higher risk should be prioritized to strengthen public policies for prevention and control.

## INTRODUCTION

Road traffic injuries (RTI) represent one of the leading causes of morbidity and mortality worldwide, generating 20 to 50 million injuries and 1.2 million deaths every year^1^. Thus, this event represents an important global public health problem and requires joint efforts to promote effective preventive measures^2^.

Brazil ranks fifth among the countries with the highest number of deaths from RTI. It is estimated that approximately 40,000 deaths from this cause occur annually in the country. Considering the serious injuries, the annual number of victims exceeds 150,000, making up total costs of around R$ 28 billion/year^2^.

The country has had high mortality rates from RTI since the 1950s. Most of these occurrences are related to the increase in the fleet of cars, the inadequacy in the adaptation of the traffic environment to accommodate it and the deficiencies in the processes of education and traffic control. With the promulgation of the Brazilian Traffic Code in 1998, more restrictive rules were put into practice and a better organization of traffic management in cities was promoted. Thus, the number of accidents and deaths resulting from RTI began to decrease, even with the continuous growth of the vehicle fleet. Between 1996 and 2000, RTI deaths fell by 17% in the country^3^.

Since then, several strategies have been implemented to curb traffic violence at the national, state and municipal levels. After the reduction period, however, the mortality rate from RTI was stable between the years 2000 and 2015. Analyzing the condition of the victim, the trend has been increasing for car occupants and motorcyclists and decreasing for pedestrians. In 2000, users with the most vulnerable condition (pedestrians, cyclists and motorcyclists) accounted for 41% of RTI deaths in Brazil. In 2015, this percentage increased to 53%, with a 400% increase in the mortality rate^4^. Thus, the harmful effects of the increase in the rate of motorization, especially the increase in mortality, were superimposed on the effect of Traffic Safety actions^5^.

It is important to emphasize that there is a great inequality among Brazilian municipalities regarding the implementation of strategies for the prevention of traffic mortality. Many are implemented only, or more rigorously, in the most populous capitals and municipalities of each state. In this sense, an important initiative is the life in transit program, initially implemented in five Brazilian capitals in 2010. Later, it was expanded to the other capitals of the country, municipalities with more than one million inhabitants and the municipalities of São José dos Pinhais and Foz do Iguaçu, in Paraná^6^^,^^7^.

Moreover, we must mention the inequalities regarding the integration of municipalities to the National Transit System (SNT). Integration is mandatory, as determined by Article 333 of the Brazilian Traffic Code (CTB)^8^, regulated by Resolution No. 560 of the National Traffic Council, of October 15, 2015. Non-integration prevents the municipality from exercising the powers set out in Article 24 of the CTB. Thus, it is prevented from implementing traffic road signs, inspecting or charging road users^9^ and from implementing proven effective measures to reduce morbidity and mortality by RTI, such as those contained in the Dry Law (Law No. 11,705, of June 19, 2008)^10^ and its revision (Law No. 12,760, of December 20, 2012)^11^.

Despite this, in 2018, of the 5,575 Brazilian municipalities, 3,982 were not yet integrated into the SNT^12^. All 27 Brazilian capitals are now integrated into the SNT. In the state of Roraima, only the capital (Boa Vista) has municipal transit^9^.

Considering that the reduction of injuries and deaths caused by traffic is one of the greatest current challenges in Brazil, studies that estimate the factors related to mortality by this cause with a greater degree of detail are essential. Such studies can identify population groups at risk and guide traffic safety interventions^13^.

Brazilian capitals have differences in population size, road structure and traffic intensity. However, they all centralize, to a greater or lesser extent, the economy of their states. When comparing capital and non-capital cities within the same state, differences of density and, in most cases, population size appear. In addition, the replacement of animal traction vehicles by motor vehicles has been observed. This change is most prominent in non-capital municipalities^13^^,^^14^. Thus, although the capitals cannot be analyzed as a single cluster, among questions related to urban mobility, the following question draws attention: are there inequalities, regarding the magnitude and trend, between the mortality by RTI in the capital and non-capital cities of each Brazilian state?

Thus, our study aims to estimate the magnitude and trend of RTI mortality according to the condition of the victim, comparing the capitals to the other municipalities of each Brazilian state, from 2000 to 2016.

## METHODS

A time series study of standardized RTI mortality rates in Brazilian municipalities was conducted from 2000 to 2016.

Data on deaths were obtained from the *Sistema de Informações sobre Mortalidade* (SIM – Mortality Information System), by the website of the Department of Informatics^15^ of the Unified Health System (Datasus)^16^. Deaths from RTI were those whose underlying cause was described in the death declaration, according to the international disease code in its tenth edition (ICD-10), with codes V01 to V89.

The annual estimates of the resident population of IBGE^17^, also collected from the Datasus website with the TabNet tool, were also used^16^. The 2016 population was estimated by the polynomial interpolation method^18^.

The annual mortality rates from RTI were estimated per 100,000 inhabitants standardized by age. This indicator was estimated according to the condition of the victim (pedestrians: V01 to V09; occupants of motorcycles or tricycles: V20 to V39; and occupants of cars or trucks: V40 to V59), municipality of residence (capital or non-capital) and year of death (2000 to 2016). Thus, it was possible to estimate the specific mortality by road traffic injuries according to the condition of the victim.

The standardization was done by the direct method, using as standard the Brazilian population in the year 2010. This method made it possible to compare the indicators both during the period and between the geographical units under study. In this sense, the results of the capitals were compared with those of the non-capital municipalities, considered as a single cluster for each state. The Federal District was excluded from the analyses because it consists of only one municipality (Brasilia).

To avoid underestimation of deaths according to the condition of the victim caused by the garbage codes, the deaths possibly related to transport accidents, but with non-specific underlying cause (V87 to V89, V99 and Y32 to Y34) were redistributed proportionally among the specific groups (pedestrians, occupants of motorcycles or tricycles, occupants of cars or trucks and other means of transport). For such purpose, the proportion of deaths, whose root cause was coded in each of these specific groups was estimated, and this proportion was applied to garbage codes. The redistribution of garbage codes related to transport accidents is recommended to correct the underestimation of rates according to accidents with specific causes^19^^,^^20^. Although the group “other means of transport” participates in the process of redistribution of deaths, this category was not analyzed to make the final value of deaths more plausible.

When the root cause of all RTI deaths occurred in a given year and municipality were described with garbage codes*,* it was not possible to perform proportional redistribution. These cases were then considered only in the analysis of all deaths due to RTI, and not in the analysis according to the condition of the victim.

This method of distribution is used by the Brazilian Ministry of Health. The flow chart with information on the number of deaths original and after redistribution is already described.

To estimate trends, the Prais-Winsten linear regression method was used. This is a method outlined for data that may be influenced by serial autocorrelation, which often occurs in population data measurements. According to Antunes and Cardoso^21^, linear autocorrelation breaks with one of the main premises of simple linear regression analysis: the independence of residues. By Prais-Winsten regression, it was possible to obtain the value of the regression slope coefficient. P = 0.05 was adopted as a critical value to determine if the trend was significant.

The average annual increase rate (AIR) was estimated using the following formula^21^:

$$Taxa\;de\;incremento\;anual=-1+10^{b},$$

*b* corresponds to the slope coefficient of the line obtained in the regression analysis relating the logarithm on a decimal basis of the mortality rate with the year of occurrence.

The 95% confidence interval of the average annual percentage increase rate in the period was estimated based on the following formula^21^:

$$IC95\%=-1+10^{\left(b\pm t\times EP\right)},$$

*t* is the value at which the Student t distribution presents 16 degrees of freedom at a two-tailed confidence level of 95% and *SE* is the standard error of *b* estimate provided by the regression analysis. Degrees of freedom are estimated by the formula *n-1*; *n* is the number of elements in the sample^21^. In our article, *n* was equal to 17, considering that a period of 17 years (2000 to 2016) was analyzed.

Regression analyses were performed using Stata 14.0 software (StataCorp. 2015. Stata Statistical Software: Release 14. College Station, TX: StataCorp LP). The estimate of the annual increase rate was performed using Microsoft Excel 2007. The graphics were developed using SPSS 25.0 software (IBM Corp. Released 2017. IBM SPSS Statistics for Windows, Version 25.0. Armonk, NY: IBM Corp.).

Submission to the Research Ethics Committee was not necessary, since this is a study that uses secondary data, without identifying the participants. Resolution No. 466 of the National Health Council of December 12, 2012 was complied with.

## RESULTS

Between 2000 and 2016, there were 485,015 deaths due to traffic injuries in Brazil. Assigned to garbage codes V87 to V89, there were 154,090 deaths, redistributed among deaths from traffic injuries with specified victim status. For V99 there were 10,598 deaths, of which 10,534 were added to the analysis. The indeterminate external causes (Y32 to Y34) had 103,354 deaths, of which 37,401 deaths were redistributed to the analysis, accounting for 202,025 garbage codes redistributed to traffic injuries. In total, 38,717 deaths were excluded, whose municipality of residence of the victim was unknown. Thus, 648,322 deaths were analyzed. In 206,338 (31.82%) of these deaths the victim was a pedestrian, in 189,994 (29.30%) the victim was an occupant of motorcycle or tricycle and in 188,569 (29.08%) the victim was an occupant of automobile or pickup truck.

Also considering the period from 2000 to 2016, among the Brazilian capitals, the highest mortality rate by standardized RTI occurred in Boa Vista, state of Roraima (RR) (41.6/100,000 inhabitants). The highest specific mortality rate of pedestrians was observed in Macapá, state of Amapá (AP) (14.7 / 100,000 inhabitants). In the condition of occupant of motorcycles or tricycles, the highest mortality also occurred in Boa Vista, RR (16.1 / 100,000 inhabitants). The highest specific rate for the condition of car occupants was found in Palmas, state of Tocantins (TO) (11.3 / 100,000 inhabitants), as shown in Figure 1.

**Figure:**
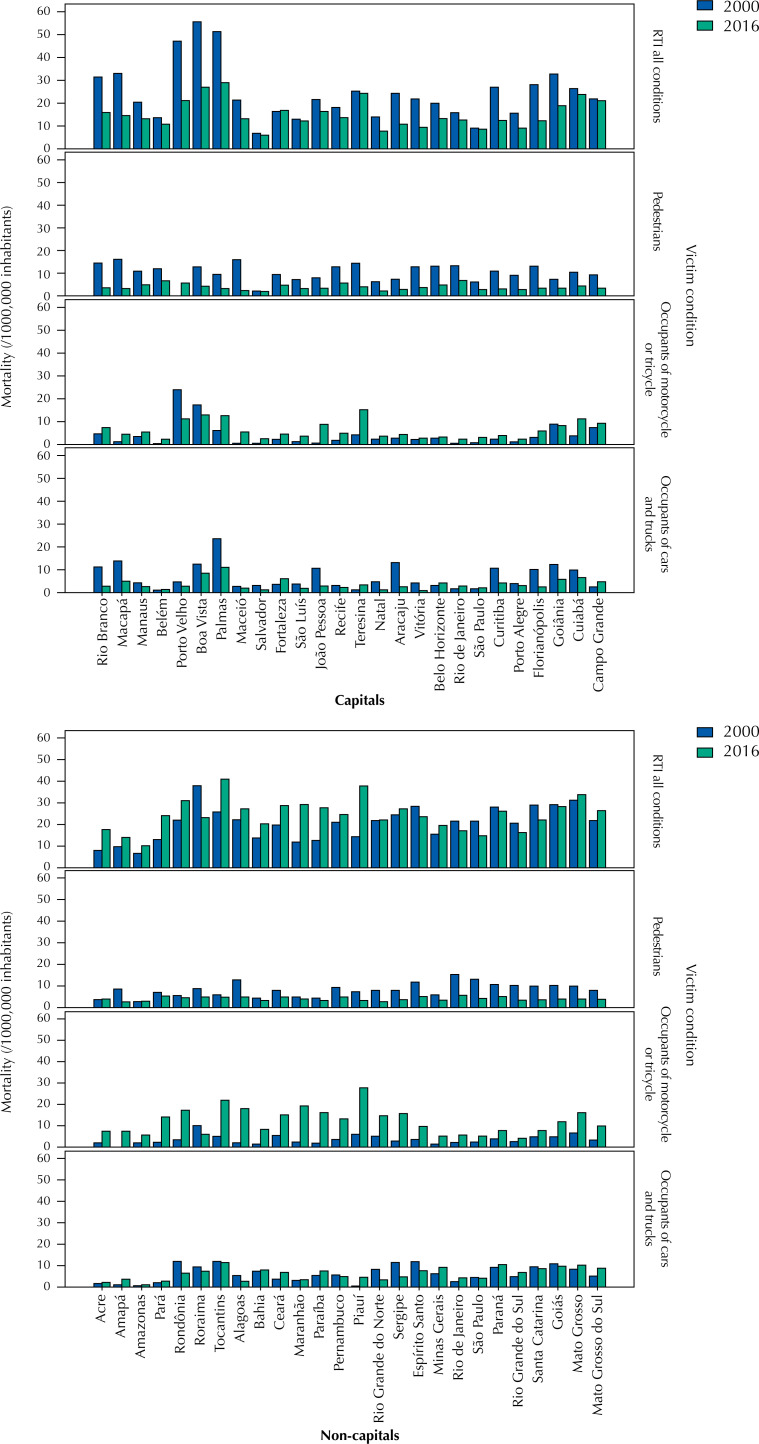
Mortality due to General Road traffic injuries and the condition of the victim according to the municipality of residence (capital and non-capital). Brazil, 2000 and 2016.

Among the non-capital municipalities, those that presented the highest mortality rate by standardized RTI were those in the state of Mato Grosso (35.6 deaths/100,000 inhabitants). Even among non-capital municipalities, the highest specific mortality rate in pedestrian condition occurred in Rio de Janeiro (10.2 / 100,000 inhabitants). In the condition of motorcycle or tricycle occupant, the highest rate was observed in the municipalities of Piauí (17.2 / 100,000 inhabitants). The highest mortality rate of a car or truck occupant occurred in the municipalities of Mato Grosso (12.1 / 100,000 inhabitants), as shown in Figure 1.

Mortality by RTI did not present an increasing trend in any of the capitals in the period. Among non-capital municipalities, an increasing trend was observed in 14 states. In the Northern region: Acre (AIR= 4.8 deaths / 100,000 inhabitants), Amazonas (AIR = 4.7 deaths / 100 thousand inhabitants), Pará (AIR = 4.1 deaths/100,000 inhabitants), Rondônia (AIR = 2.1 deaths/ 100,000 inhabitants) and Tocantins (AIR = 3.5 deaths/ 100,000 inhabitants). In the Northeast region, all states, except Rio Grande do Norte, showed an increasing trend of mortality by RTI in non-capital cities. In the Southeast and South regions, there was no increasing trend of mortality in the states under study. In the Midwest Region, there was an increasing trend only in Mato Grosso (AIR = 1.0 death/ 100,000 inhabitants), as shown in Table 1.

**Table 1:** Annual increase rate of mortality by RTI according to the city of residence of the victim. Brazil, 2000–2016.

Region	Capitals	Mortality by RTI				Non-capitals*	Mortality by RTI			
		AIR	LL	UL	Tendency		AIR	LL	UL	Tendency
North	Rio Branco	-3.3	-6.1	-0.5	↓	AC	4.8	1.8	7.9	↑
	Macapá	-4.4	-6.6	-2.2	↓	AP	1.0	-1.1	3.2	–––
	Manaus	-1.7	-3.2	-0.2	↓	AM	4.7	2.5	6.9	↑
	Belém	-1.1	-2.3	0.1	–––	PA	4.1	3.2	4.9	↑
	Porto Velho	-2.5	-5.1	0.1	–––	RO	2.1	0.2	3.9	↑
	Boa Vista	-2.4	-4.2	-0.6	↓	RR	0.3	-1.8	2.4	–––
	Palmas	-1.4	-2.1	-0.6	↓	TO	3.5	2.4	4.6	↑
	Maceió	-2.3	-3.6	-1.1	↓	AL	2.5	1.0	3.9	↑
	Salvador	0.4	-2.6	3.5	–––	BA	3.2	1.3	5.1	↑
	Fortaleza	-0.6	-2.1	0.9	–––	CE	3.4	1.9	4.9	↑
	São Luís	-0.2	-2.3	1.9	–––	MA	6.4	5.0	7.9	↑
Northeast	João Pessoa	-2.6	-3.5	-1.7	↓	PB	5.2	4.0	6.5	↑
	Recife	-1.0	-2.7	0.8	–––	PE	1.6	0.1	3.0	↑
	Teresina	0.7	-0.6	2.1	–––	PI	7.5	5.5	9.6	↑
	Natal	-3.3	-4.3	-2.1	↓	RN	0.7	-0.3	1.6	–––
	Aracaju	-3.3	-6.3	-0.2	↓	SE	1.7	0.0	3.4	↑
Southeast	Vitória	-3.9	-5.4	-2.5	↓	ES	-0.6	-2.5	1.3	–––
	Belo Horizonte	-2.6	-5.1	0.1	–––	MG	1.5	-0.8	4.0	–––
	Rio de Janeiro	-1.8	-3.7	0.2	–––	RJ	-1.4	-3.0	0.3	–––
	São Paulo	-1.6	-3.8	0.7	–––	SP	-2.3	-3.3	-1.3	↓
South	Curitiba	-4.8	-6.6	-2.9	↓	PR	-0.3	-2.2	1.7	–––
	Porto Alegre	-3.4	-4.6	-2.3	↓	RS	-1.2	-2.4	0.0	↓
	Florianópolis	-4.2	-5.5	-2.8	↓	SC	-1.7	-3.8	0.4	–––
Midwest	Goiânia	-2.9	-4.2	-1.5	↓	GO	0.7	-0.2	1.6	–––
	Cuiabá	-0.1	-1.7	1.6	–––	MT	1.0	0.0	2.1	↑
	Campo Grande	-1.1	-2.9	0.6	–––	MS	1.3	-1.2	3.8	–––

* Non-capital municipalities of each state.

RTI: road traffic injuries; AIR: annual increment rate; LL: lower limit of 95% confidence interval; UL: upper limit of 95% confidence interval.

The specific mortality rate for pedestrians did not show an increasing trend in any of the states analyzed. However, rates were stable in four capitals of the Northern Region (Macapá, Porto Velho, Boa Vista and Palmas) and one capital of the Northeast Region (Salvador). In nine states of the North and Northeast regions, rates were stable in non-capital municipalities. In the Midwest, Southeast and South regions, specific mortality in pedestrian condition showed a decreasing trend in all analyses (Table 2).

**Table 2:** Annual increase rate of mortality by RTI according to the city of residence of the victim. Brazil, 2000–2016.

Region	Capitals	Mortality of pedestrians				Non-capitals*	Mortality of pedestrians			
		AIR	LL	UL	Tendency		AIR	LL	UL	Tendency
North	Rio Branco	-9.3	-12.9	-5.6	↓	AC	-1.6	-6.7	3.8	–––
	Macapá	-11.1	-22.4	1.8	–––	AP	-8.2	-13.8	-2.2	↓
	Manaus	-4.2	-8.1	-0.1	↓	AM	1.3	-1.5	4.2	–––
	Belém	-2.6	-4.4	-0.8	↓	PA	0.0	-1.8	1.9	–––
	Porto Velho	5.9	-3.4	16.0	–––	RO	-2.2	-3.5	-1.0	↓
	Boa Vista	-3.8	-9.8	2.7	–––	RR	2.2	-2.1	6.7	–––
	Palmas	-2.8	-7.6	2.3	–––	TO	-0.8	-3.0	1.5	–––
Northeast	Maceió	-7.7	-10.6	-4.6	↓	AL	-4.1	-6.2	-2.0	↓
	Salvador	-1.7	-5.7	2.5	–––	BA	-1.2	-3.4	1.1	–––
	Fortaleza	-3.2	-5.7	-0.6	↓	CE	-1.6	-4.3	1.2	–––
	São Luís	-5.1	-7.7	-2.3	↓	MA	-0.4	-3.0	2.2	–––
	João Pessoa	-7.1	-8.9	-5.2	↓	PB	-5.8	-8.1	-3.5	↓
	Recife	-4.4	-6.2	-2.6	↓	PE	-3.7	-4.8	-2.6	↓
	Teresina	-5.7	-6.4	-5.0	↓	PI	-1.9	-4.1	0.4	–––
	Natal	-6.4	-11.2	-1.3	↓	RN	-5.8	-7.4	-4.2	↓
	Aracaju	-5.3	-8.4	-2.0	↓	SE	-4.4	-6.7	-2.0	↓
Southeast	Vitória	-5.7	-7.4	-3.9	↓	ES	-5.6	-7.2	-4.0	↓
	Belo Horizonte	-6.3	-9.5	-3.1	↓	MG	-3.4	-5.3	-1.4	↓
	Rio de Janeiro	-3.8	-5.6	-2.0	↓	RJ	-5.4	-7.2	-3.7	↓
	São Paulo	-5.9	-8.0	-3.9	↓	SP	-7.8	-9.4	-6.1	↓
South	Curitiba	-7.4	-8.8	-5.9	↓	PR	-4.7	-6.4	-3.0	↓
	Porto Alegre	-7.2	-8.9	-5.4	↓	RS	-6.9	-7.8	-6.0	↓
	Florianópolis	-8.1	-10.4	-5.8	↓	SC	-6.7	-8.3	-5.2	↓
Midwest	Goiânia	-5.6	-7.9	-3.3	↓	GO	-5.1	-7.3	-3.0	↓
	Cuiabá	-5.7	-9.0	-2.2	↓	MT	-4.4	-5.7	-3.1	↓
	Campo Grande	-9.6	-11.6	-7.6	↓	MS	-5.8	-6.9	-4.6	↓

* Non-capital municipalities of each state.

RTI: road traffic injuries; AIR: annual increment rate; LL: lower limit of 95% confidence interval; UL: upper limit of 95% confidence interval.

The mortality rate of occupants of motorcycles or tricycles did not show a decreasing trend in any of the states. There was a steady trend in rates in 10 capitals. In the Northern Region: Rio Branco, Porto Velho and Boa Vista. In The Northeast Region: Aracaju. In the Southeast Region: Vitória and Belo Horizonte. In The Midwest Region: Goiânia. In the Southern Region, all capitals showed a steady trend. Considering the non-capital municipalities, only four states showed a steady trend in mortality rates for motorcycle or tricycle occupants: Roraima, Paraná, Rio Grande do Sul and Santa Catarina (Table 3).

**Table 3:** Annual increase rate of mortality of motorcycle/tricycle occupants according to the city of residence of the victim. Brazil, 2000–2016.

Region	Capitals	Mortality of motorcycle/tricycle occupants				Non-capitals*	Mortality of motorcycle/tricycle occupants			
		AIR	LL	LU	Tendency		AIR**	LL	LU	Tendency
North	Rio Branco	3.1	-9.8	17.8	–––	AC	12.1	6.6	17.8	↑
	Macapá	14.0	7.5	20.9	↑	AP	16.6	10.5	23.1	↑
	Manaus	4.7	2.1	7.4	↑	AM	8.5	5.3	11.8	↑
	Belém	14.9	10.6	19.3	↑	PA	11.7	10.4	12.9	↑
	Porto Velho	-3.2	-9.1	3.0	–––	RO	6.8	3.2	10.6	↑
	Boa Vista	0.6	-5.3	6.8	–––	RR	2.1	-3.4	7.8	–––
	Palmas	2.7	0.4	5.0	↑	TO	9.1	7.1	11.2	↑
Northeast	Maceió	13.9	7.4	20.9	↑	AL	14.5	9.7	19.6	↑
	Salvador	17.3	10.6	24.5	↑	BA	12.8	11.1	14.7	↑
	Fortaleza	4.5	0.2	9.1	↑	CE	7.8	5.3	10.4	↑
	São Luís	9.8	6.3	13.4	↑	MA	15.2	12.8	17.6	↑
	João Pessoa	12.9	7.4	18.7	↑	PB	15.0	8.3	22.1	↑
	Recife	8.8	5.1	12.7	↑	PE	9.8	7.2	12.4	↑
	Teresina	7.9	5.7	10.1	↑	PI	11.1	8.2	14.0	↑
	Natal	4.9	0.5	9.6	↑	RN	7.6	5.6	9.6	↑
	Aracaju	3.5	-3.9	11.5	–––	SE	11.7	6.4	17.3	↑
Southeast	Vitória	2.5	-1.7	6.9	–––	ES	6.9	2.5	11.4	↑
	Belo Horizonte	2.3	-1.7	6.4	–––	MG	8.4	3.4	13.7	↑
	Rio de Janeiro	9.8	0.5	20.0	↑	RJ	6.3	2.0	10.9	↑
	São Paulo	8.6	3.4	14.0	↑	SP	5.0	0.8	9.3	↑
South	Curitiba	3.1	-2.1	8.6	–––	PR	4.6	-0.2	9.6	–––
	Porto Alegre	2.9	-0.3	6.3	–––	RS	3.4	-1.5	8.6	–––
	Florianópolis	2.8	-0.7	6.5	–––	SC	3.2	-1.8	8.5	–––
Midwest	Goiânia	0.1	-2.2	2.5	–––	GO	6.0	4.0	8.0	↑
	Cuiabá	8.7	6.3	11.1	↑	MT	6.3	2.8	9.9	↑
	Campo Grande	5.1	2.5	7.8	↑	MS	7.5	2.6	12.7	↑

* Non-capital municipalities of each state.

RTI: road traffic injuries; AIR: annual increment rate; LL: lower limit of 95% confidence interval; UL: upper limit of 95% confidence interval.

Of the Capitals, the only one that showed an increasing trend of mortality of occupants of cars or trucks was Teresina (AIR = 9.5 deaths/100,000 inhabitants). Among the non-capital municipalities, nine showed an increasing trend. In the Northern Region: Amazonas (AIR = 5.8 deaths/100,000 inhabitants), Pará (AIR = 1.5 deaths/100,000 inhabitants) and Tocantins (AIR = 2.4 deaths/100,000 inhabitants). In the Northeast Region: Ceará (AIR = 2.9 deaths/100,000 inhabitants), Paraíba (AIR = 5.1 deaths/100,000 inhabitants) and Piauí (AIR = 14.0 deaths/100,000 inhabitants). In the Southeast Region: Minas Gerais (AIR = 2.6 deaths/100,000 inhabitants). In the Southern Region: Rio Grande do Sul (AIR = 2.1 deaths/100,000 inhabitants). In the Midwest Region: Mato Grosso do Sul (AIR = 4.6 deaths/100,000 inhabitants). There was a decrease in the mortality of occupants of cars or trucks in six capitals. In the Northeast: Natal (AIR = −4.3 deaths/100,000 inhabitants) and Aracaju (AIR = −6.7 deaths/100,000 inhabitants). In the Southern Region: Curitiba (AIR = −5.4 deaths/100,000 inhabitants) and Florianópolis (AIR = −5.5 deaths/100,000 inhabitants). In the Midwest Region: Goiânia (AIR = −3.1 deaths/100,000 inhabitants) and Cuiabá (AIR = −1.9 deaths/100,000 inhabitants). Among the non-capital municipalities, there was a decrease in the rate only in Rio Grande do Norte (AIR = −4.3 deaths/100,000 inhabitants) and Sergipe (AIR = −3.7 deaths/100,000 inhabitants), both in the Northeast Region. The other stated under analysis showed a steady trend of specific mortality rates of car or truck occupants in the period (Table 4).

**Table 4:** Annual increase rate of mortality of occupants of cars or trucks according to the city of residence of the victim. Brazil, 2000–2016.

Region	Capitals	Mortality of car and truck occupants				Non-capitals*	Mortality of car and truck occupants			
		AIR	LL	LU	Tendency		AIR	LL	LU	Tendency
North	Rio Branco	-1.0	-11.4	10.7	–––	AC	1.8	-5.1	9.3	–––
	Macapá	1.9	-7.5	12.1	–––	AP	3.9	-3.1	11.5	–––
	Manaus	-1.4	-4.2	1.4	–––	AM	5.8	1.6	10.2	↑
	Belém	-2.6	-8.5	3.6	–––	PA	1.5	0.5	2.5	↑
	Porto Velho	-0.4	-11.3	11.8	–––	RO	-0.4	-4.0	3.3	–––
	Boa Vista	-3.9	-7.9	0.2	–––	RR	-1.4	-8.1	5.9	–––
	Palmas	-2.1	-6.0	2.0	–––	TO	2.4	0.8	4.1	↑
Northeast	Maceió	-1.1	-5.1	3.1	–––	AL	-1.1	-5.8	3.8	–––
	Salvador	-4.0	-9.4	1.8	–––	BA	1.0	-1.6	3.7	–––
	Fortaleza	-0.5	-3.2	2.3	–––	CE	2.9	1.2	4.7	↑
	São Luís	-0.8	-6.7	5.5	–––	MA	1.1	-1.2	3.5	–––
	João Pessoa	-4.7	-9.9	0.9	–––	PB	5.1	1.8	8.5	↑
	Recife	0.6	-4.4	5.9	–––	PE	0.4	-0.7	1.4	–––
	Teresina	9.5	5.8	13.2	↑	PI	14.0	8.7	19.6	↑
	Natal	-6.7	-10.6	-2.6	↓	RN	-4.3	-5.3	-3.3	↓
	Aracaju	-6.7	-12.0	-1.0	↓	SE	-3.7	-6.6	-0.7	↓
Southeast	Vitória	-9.4	-19.5	1.9	–––	ES	-1.3	-3.8	1.2	–––
	Belo Horizonte	0.9	-1.9	3.8	–––	MG	2.6	0.3	4.9	↑
	Rio de Janeiro	-1.2	-6.1	3.9	–––	RJ	2.2	-1.3	5.9	–––
	São Paulo	-0.1	-2.1	1.9	–––	SP	0.0	-2.5	2.6	–––
South	Curitiba	-5.4	-7.2	-3.6	↓	PR	1.1	-0.5	2.7	–––
	Porto Alegre	-1.0	-3.2	1.3	–––	RS	2.1	0.9	3.2	↑
	Florianópolis	-5.5	-7.1	-3.8	↓	SC	-0.6	-1.7	0.5	–––
Midwest	Goiânia	-3.1	-5.7	-0.3	↓	GO	0.5	-2.0	3.1	–––
	Cuiabá	-1.9	-3.8	0.0	↓	MT	-0.6	-2.7	1.6	–––
	Campo Grande	0.1	-3.5	3.9	–––	MS	4.6	1.2	8.0	↑

* Non-capital municipalities of each state.

RTI: road traffic injuries; AIR: annual increment rate; LL: lower limit of 95% confidence interval; UL: upper limit of 95% confidence interval.

## DISCUSSION

From 2000 to 2016, non-capital cities presented unfavorable trends in standardized mortality rates by RTI compared to the capitals of their respective states. This discrepancy was most pronounced in the poorest macro-regions of the country (North and Northeast). Accidents involving motorcycles were the category, in which a more pronounced increasing trend was observed, which reflects the internalization of the use of this type of vehicle in the country.

There was a preponderance of a steady trend (observed in 12 capitals and 10 non-capital clusters) or a reduction (observed in 14 capitals and 2 non-capital clusters) of mortality rates by RTI in Brazil in the period. This trend may have been caused by improvements in road infrastructure, a reduction in average speed (both by increasing the flow of vehicles and by implementing speed control and surveillance measures), an increase in the use of safety equipment (helmet, seat belt, child control equipment, etc.), decreased risk factors such as “drinking and driving” and also by improving pre-hospital and hospital care for victims^13^. The reduction in the magnitude of pedestrian mortality follows a worldwide trend and is related to these same factors^2^^,^^22^. The reduction can also be explained by the expansion of traffic education campaigns and the emphasis on the use of the pedestrian crossing.^23^

According to the Statistical Yearbook of Personal Injury Insurance caused by land-based motor vehicles, there was a reduction in the number of compensation paid for deaths in traffic accidents throughout Brazil since 2012. Souza et al.^24^ reported an increase in the standardized mortality rate from motorcycle accidents and hit-and-run accidents in the period from 1980 to 2003 in Brazil. In the same period, the authors observed a reduction in mortality due to accidents involving other means of land transport. Morais Neto et al.^22^ reported that the mortality rate from RTI in Brazil ranged from 18.2 per 100,000 inhabitants in 2000 to 22.54 per 100,000 inhabitants in 2010, representing an increase of 22.54%. Scolari et al.^25^ observed a reduction in the RTI mortality rate in Brazil between 2006 and 2008, both considering as denominator the number of inhabitants and considering the vehicle fleet.

The marked increase in mortality of motorcyclists, especially in the North, Northeast and Midwest regions, may be related to the rapid growth of the vehicle fleet. Although it occurred throughout the country, it was most highlighted in the three regions cited^7^^,^^26^^–^^29^. Mainly in inland municipalities, the motorcycle has been used as a replacement for non-motorized vehicles, such as bicycles and animal-traction vehicles^13^.

To the vulnerability of motorcyclists' body exposure, the risk behavior adopted by them is added, which considerably increases the risks of injuries and deaths. The 2013 National Health Survey showed that, in the North, Northeast and Midwest regions, helmet use is less frequent than in other regions. Wearing a helmet reduces the risk of death by 40% and the risk of injury by 70%. Moreover, the North and Northeast regions have the lowest percentage of municipalities included in the National Transit System. As a consequence, they have lower surveillance capacity and investment in signaling and maintenance of road infrastructure^30^.

The inequality between RTI mortality trends in capitals and non-capital municipalities of each state between 2000 and 2016 can be explained by the unequal increase in the fleet and the unequal capacity to implement regulatory measures in traffic. The ease of acquisition of motor vehicles, especially motorcycles, was favored by the tax benefits implemented by the federal government both with regard to their production and consumption^25^. In addition, public transport is inefficient or even inexistent in many municipalities, and urban mobility policies favor individual and private transport over public transport^26^. These factors, associated with economic growth, caused the motor vehicle fleet in Brazil to increase from 29,722,950 to 93,867,016 between 2000 and 2016. Motorcycles, which corresponded to 11.90% of the total national fleet at the beginning of this period, represented 22.31% in 2016, remaining 22.22% in 2018^27^.

In recent decades, there has been an increasing strengthening of legislation related to the Prevention of risk factors for traffic deaths and injuries. The Brazilian Traffic Code was instituted in 1998, seeking to circumscribe the main Behavioral Risk Factors for RTI (speed, driving after drinking alcohol and not using the helmet, seat belt and device for transporting children)^31^^,^^32^. However, its progressive implementation in the following years occurred unevenly among the capitals and non-capital municipalities, as well as among the macro-regions, according to their differential conditions for implementing and supervising the transit system^33^. Thus, its possible favorable effects for the reduction of RTI mortality also occurred unevenly and the results described here are compatible with this hypothesis^34^.

The surveillance capacity of the SNT bodies, for example, is still insufficient in the three spheres of management of the country^7^. In the municipal sphere, only 28.22% of non-capital municipalities are part of the SNT. Meanwhile, 100% of the capitals adhered to the municipalization of traffic^11^. The comparison between the mortality rates from RTI in the years 2000 and 2015 showed lower increase in the mortality rate among municipalities with traffic integrated to the SNT (percentage variation equal to 8.5%) compared to those with non-integrated traffic (29.1%)^13^.

In 2001, the National Policy for the reduction of morbidity and mortality due to accidents and violence was created (ordinance MS/GM No. 737 of May 16, 2001), establishing the need for interventions that promote the adoption of safe and healthy behaviors and environments. In 2004, the National Transit Policy was published (Resolution No. 166, of September 15, 2004), which included traffic in the global government plan and presented objectives, guidelines and targets for its improvement in Brazil. In 2010, The Life In Traffic project was created, coordinated by the Brazilian Ministry of Health. The project presupposed priority intervention in two risk factors for traffic accidents: excessive and inadequate speed and driving vehicles after drinking alcohol. The project became the *Programa Vida no Trânsito* (Life in Traffic Program), expanded in 2013^6^. Moreover, in 2011, the National Traffic Department implemented the National Pact for the Reduction of Traffic Accidents, with the participation of the Ministry of Health and the Ministry of Cities^35^. All these measures probably contributed to the decline of mortality indicators by RTI, especially in the richest regions of the country and in the Capitals, which have more resources and possibly managed to implement them more quickly and extensively in their territories^36^. This could also explain the differences in mortality among means of transport, among capital and non-capital cities and among macro-regions. In the case of mortality of motorcycle occupants, the marked increase in the fleet overlapped with the aforementioned public policies^37^. Thus, mortality by this cause was increasing in most of the units analyzed.

The lack of information on the implementation differentials of the Brazilian Traffic Code among cities and among macro-regions is a limitation of our study. The fact that there are quality differences in the identification of the causes of death can also be considered a limitation of the study^38^. In the Mortality Information System, there is the possibility of underreporting the causes of death, especially in small municipalities and in the poorest macro-regions of the country, which may partially affect the results in an uncontrolled way.

The results of our article suggest that public accident prevention policies have not been as effective as market policies for increasing vehicle sales. Permanent monitoring of compliance with traffic legislation and investments in traffic engineering are fundamental actions to reverse this framework. Actions related to urban mobility should also be implemented through the improvement of the quality and frequency of public transport and the construction of adequate cycle crossings. These measures need to be implemented evenly in municipalities and regions to reduce the inequalities we observed.
