# Supplementary material for: Mortality by road traffic injuries in Brazil (2000–2016): capital cities versus non-capital cities
Source: Rev Saude Publica. 2020 Nov 17;54:122. doi: 10.11606/s1518-8787.2020054001703 (PMC7671585; doi:10.11606/s1518-8787.2020054001703)
Supplement: Translation

# Translation: Mortalidade por acidentes de trânsito no Brasil (2000–2016): capitais versus não capitais

**Authors:** Érika Carvalho de Aquino, José Leopoldo Ferreira Antunes, Otaliba Libânio de Morais

**Affiliations:** I GoiâniaGO Brasil Universidade Federal de Goiás. Instituto de Patologia Tropical e Saúde Pública. Programa de Pós-Graduação em Medicina Tropical e Saúde Pública. Goiânia, GO, Brasil; II São PauloSP Brasil Universidade de São Paulo. Faculdade de Saúde Pública. Departamento de Epidemiologia. São Paulo, SP, Brasil; III GoiâniaGO Brasil Universidade Federal de Goiás. Instituto de Patologia Tropical e Saúde Pública. Departamento de Epidemiologia. Goiânia, GO, Brasil

**Keywords:** Acidentes de Trânsito, mortalidade, Análise Espacial, Estudos de Séries Temporais

## Abstract

**OBJETIVO:**

Comparar a magnitude e tendência da mortalidade por acidentes de transporte terrestre (ATT) nas capitais e demais municípios de cada estado brasileiro nos anos de 2000 a 2016.

**MÉTODOS:**

Foi realizada análise de séries temporais das taxas de mortalidade por ATT padronizadas por idade, comparando as capitais e o aglomerado de municípios não capitais em cada estado. Os dados sobre óbitos foram obtidos do Sistema de Informações sobre Mortalidade. Foram considerados como óbitos por ATT aqueles cuja causa básica tenha sido designada pelos códigos V01 a V89 do CID-10, com redistribuição dos *garbage codes*. Para o cálculo das taxas de mortalidade, foram utilizadas as projeções populacionais do Instituto Brasileiro de Geografia e Estatística de 2000 a 2015 e a população calculada por interpolação polinomial para 2016. A análise de tendências foi realizada pelo método de Prais-Winsten, utilizando o programa Stata 14.0.

**RESULTADOS:**

Ocorreram 601.760 óbitos por ATT no período (114.483 de residentes em capitais). A mortalidade por ATT não apresentou tendência crescente em nenhuma das capitais no período em estudo. Já entre os municípios não capitais, a tendência foi crescente em 14 estados. O maior aumento foi observado no Piauí (TIA = 7,50%; IC95% 5,50 – 9,60). Ocorreu tendência decrescente da mortalidade por ATT em 14 capitais, dentre as quais Curitiba apresentou maior decréscimo (TIA = −4,82%; IC95% −6,61 – −2,92). Apenas São Paulo e Rio Grande do Sul apresentaram tendência decrescente da mortalidade por ATT nos municípios não capitais (TIA = 2,32%; IC95% −3,32 – −1.3 e TIA = 1,2%, IC95% −2,41 – 0,00, respectivamente).

**CONCLUSÕES:**

É possível concluir que as taxas de mortalidade por ATT em municípios não capitais no Brasil apresentaram tendência alarmantes quando comparadas às observadas nas capitais. O desenvolvimento de ações eficazes de segurança no trânsito está quase sempre limitado às capitais e grandes cidades brasileiras. Os municípios com maior risco devem ser priorizados para o fortalecimento das políticas públicas de prevenção e controle.

## INTRODUÇÃO

Os acidentes de transporte terrestre (ATT) representam uma das principais causas de morbidade e mortalidade em todo o mundo, gerando de 20 a 50 milhões de lesões e 1,2 milhão de mortes todos os anos^1^. Dessa maneira, esse evento representa um importante problema de saúde pública global e demanda esforços conjuntos para que se promovam medidas preventivas eficazes^2^.

O Brasil ocupa a quinta posição entre os países com maior número de mortes por ATT. Estima-se que ocorram, ao ano, cerca de 40 mil óbitos por essa causa em todo o território nacional. Considerando os feridos graves, o número anual de vítimas ultrapassa 150 mil, perfazendo custos totais que orbitam em torno de R$ 28 bilhões/ano^2^.

O país vem apresentando altas taxas de mortalidade por ATT desde a década de 1950. Grande parte dessas ocorrências está relacionada ao aumento da frota de automóveis, à insuficiência na adaptação do ambiente de circulação para acolhê-la e às deficiências nos processos de educação e fiscalização no trânsito. Com a promulgação do Código de Trânsito Brasileiro, em 1998, foram colocadas em prática regras mais restritivas e promoveu-se uma melhor organização da gestão do trânsito nas cidades. Dessa forma, o número de acidentes e mortes decorrentes de ATT começou a diminuir, mesmo com o contínuo crescimento da frota de veículos. Entre 1996 e 2000, as mortes por ATT no país caíram 17%^3^.

Foram implementadas, desde então, diversas estratégias para o enfretamento da violência no trânsito em nível nacional, estadual e municipal. Após o período de redução, entretanto, foi observada estabilidade da taxa de mortalidade por ATT entre os anos de 2000 e 2015. Analisando a condição da vítima, a tendência foi crescente para ocupantes de automóvel e motociclistas, enquanto para pedestres foi decrescente. Em 2000, os usuários com condição mais vulnerável (pedestres, ciclistas e motociclistas) representaram 41% dos óbitos por ATT no Brasil. Em 2015, esse percentual subiu para 53%, com um aumento de 400% na taxa de mortalidade^4^. Dessa forma, os efeitos danosos do crescimento da taxa de motorização, em especial o aumento da mortalidade, se sobrepuseram ao efeito das ações de segurança no trânsito^5^.

É importante ressaltar que existe uma grande desigualdade entre os municípios brasileiros no que diz respeito à implementação das estratégias para a prevenção da mortalidade no trânsito. Muitas são implementadas apenas, ou de maneira mais rigorosa, nas capitais e municípios mais populosos de cada estado. Nesse sentido, uma iniciativa importante é o Programa Vida no Trânsito, implantado inicialmente em cinco capitais brasileiras no ano de 2010. Posteriormente foi expandido para as demais capitais do país, municípios com mais de um milhão de habitantes e os municípios de São José dos Pinhais e Foz do Iguaçu, no Paraná^6^^,^^7^.

Além disso, há que se citar as desigualdades quanto à integração dos municípios ao Sistema Nacional de Trânsito (SNT). A integração é obrigatória, conforme determina o artigo 333 do Código de Trânsito Brasileiro (CTB)^8^, regulamentado pela Resolução n° 560 do Conselho Nacional de Trânsito, de 15 de outubro de 2015. A não integração impede que o município exerça as competências estabelecidas no artigo 24 do CTB. Assim, ele fica impedido de implantar sinalização viária de trânsito, fiscalizar ou autuar usuários das vias^9^ e de implementar medidas comprovadamente efetivas na redução da morbimortalidade por ATT, como as contidas na Lei Seca (Lei n° 11.705, de 19 de junho de 2008)^10^ e sua revisão (Lei n° 12.760, de 20 de dezembro de 2012)^11^.

Apesar disso, em 2018, dos 5.575 municípios brasileiros, 3.982 ainda não estavam integrados ao SNT^12^. Já todas as 27 capitais brasileiras estão, hoje, integradas ao SNT. Em Roraima, apenas a capital (Boa Vista) possui trânsito municipalizado^9^.

Considerando que a redução das lesões e mortes causadas pelo trânsito é um dos maiores desafios atuais do Brasil, estudos que estimem com maior grau de detalhamento os fatores relacionados à mortalidade por essa causa são essenciais. Tais estudos podem identificar grupos populacionais de risco e orientar intervenções de segurança no trânsito^13^.

As capitais brasileiras guardam diferenças entre si no que diz respeito ao porte populacional, estrutura viária e intensidade do tráfego. Entretanto, a elas é comum o fato de centralizarem, em maior ou menor escala, a economia de seus estados. Ao compararmos municípios capitais e não capitais dentro de um mesmo estado, é possível observar diferenças no que diz respeito à densidade e, na maior parte dos casos, porte populacional. Além disso, vem sendo observada, nos últimos anos, a substituição de veículos de tração animal por veículos motorizados. Essa mudança é mais proeminente nos municípios não capitais^13^^,^^14^. Assim, há questões relacionadas à mobilidade urbana que fazem com que, embora as capitais não possam ser analisadas como um aglomerado único, diferentes que são, a seguinte pergunta chame a atenção: existem desigualdades, quanto à magnitude e tendência, entre a mortalidade por ATT observada nos municípios capitais e não capitais de cada estado brasileiro?

Dessa maneira, o presente trabalho tem por objetivo estimar a magnitude e tendência da mortalidade por ATT segundo condição da vítima, comparando as capitais aos demais municípios de cada estado brasileiro, nos anos de 2000 a 2016.

## MÉTODOS

Foi realizado um estudo das séries temporais das taxas padronizadas de mortalidade por ATT nos municípios brasileiros, no período de 2000 a 2016.

Os dados sobre óbitos foram obtidos a partir do Sistema de Informações sobre Mortalidade (SIM)^15^, pelo site do Departamento de Informática do Sistema Único de Saúde (Datasus)^16^. Foram considerados como óbitos por ATT aqueles cuja causa básica tenha sido descrita na declaração de óbito, de acordo com o Código Internacional de Doenças em sua décima edição (CID-10), com os códigos V01 a V89.

Foram utilizadas, ainda, as estimativas anuais de população residente do IBGE^17^, também coletadas a partir do site do Datasus por meio da ferramenta TabNet^16^. A população de 2016 foi estimada pelo método de interpolação polinomial^18^.

Foram calculadas as taxas anuais de mortalidade por ATT a cada 100 mil habitantes padronizadas por idade. Esse indicador foi calculado segundo condição da vítima (pedestres: V01 a V09; ocupantes de motocicletas ou triciclos: V20 a V39; e ocupantes de automóveis ou caminhonetes: V40 a V59), município de residência (capital ou não capital) e ano do óbito (2000 a 2016). Dessa maneira, foi possível calcular a mortalidade específica por acidentes de transporte terrestre segundo a condição da vítima.

A padronização foi feita pelo método direto, utilizando como padrão a população brasileira no ano de 2010. Esse método possibilitou a comparabilidade dos indicadores tanto no decorrer do período quanto entre as unidades geográficas em estudo. Nesse sentido, foram comparados os resultados das capitais àqueles dos municípios não capitais, considerados como um aglomerado único para cada estado. O Distrito Federal foi excluído das análises por ser composto por apenas um município (Brasília).

Para evitar subestimativa de óbitos segundo a condição da vítima, ocasionada pelos códigos pouco úteis (“*garbage codes*”), os óbitos possivelmente relacionados a acidentes de transporte, mas com causa básica não específica (V87 a V89, V99 e Y32 a Y34) foram redistribuídos proporcionalmente entre os grupos específicos (pedestres, ocupantes de motocicletas ou triciclos, ocupantes de automóveis ou caminhonetes e outros meios de transporte). Para tanto, foi calculada a proporção de óbitos cuja causa básica tenha sido codificada em cada um desses grupos específicos, e essa proporção foi aplicada aos *garbage codes*. A redistribuição dos *garbage codes* relacionados aos acidentes de transporte é recomendada para correção da subestimativa das taxas segundo acidentes com causas específicas^19^^,^^20^. Embora o grupo “outros meios de transporte” participe do processo de redistribuição dos óbitos, de modo a tornar o valor final de óbitos mais verossímil, essa categoria não foi analisada.

Nos casos em que a causa básica de todos os óbitos por ATT ocorridos em determinado ano e município estavam descritos com *garbage codes*, não foi possível realizar a redistribuição proporcional. Esses casos foram, então, considerados apenas na análise da totalidade dos óbitos por ATT, e não nas análises segundo condição da vítima.

Esse método de distribuição é utilizado pelo Ministério da Saúde. O fluxograma com as informações sobre o número de óbitos original e após redistribuição já se encontra descrito.

Para estimar as tendências, foi utilizado método de regressão linear de Prais-Winsten. Este é um método delineado para dados que possam ser influenciados pela autocorrelação serial, o que frequentemente ocorre em medidas de dados populacionais. Segundo Antunes e Cardoso^21^, a autocorrelação linear rompe com uma das principais premissas da análise de regressão linear simples: a independência dos resíduos. Por meio da regressão de Prais-Winsten, foi possível obter o valor do coeficiente de inclinação da regressão. Foi adotado como valor crítico para determinar se a tendência foi significativa o p = 0,05.

A taxa de incremento médio anual (TIA) foi calculada utilizando a seguinte fórmula^21^:

$$Taxa\;de\;incremento\;anual=-1+10^{b},$$

onde *b* corresponde ao coeficiente de inclinação da reta obtida na análise de regressão relacionando o logaritmo em base decimal da taxa de mortalidade com o ano de ocorrência.

O intervalo de 95% de confiança da taxa de incremento médio anual percentual no período foi calculado a partir da seguinte fórmula^21^:

$$IC95\%=-1+10^{\left(b\pm t\times EP\right)},$$

onde *t* é o valor em que a distribuição t de Student apresenta 16 graus de liberdade a um nível de confiança de 95% bicaudal e *EP* é o erro padrão da estimativa de *b* fornecido pela análise de regressão. Os graus de liberdade são calculados por meio da fórmula *n-1*, onde *n* é o número de elementos na amostra^21^. Neste trabalho, o valor de *n* foi igual a 17, tendo em vista que foi analisado um período de 17 anos (2000 a 2016).

As análises de regressão foram executadas utilizando o *software* Stata 14.0 (StataCorp. 2015. *Stata Statistical Software: Release 14*. College Station, TX: StataCorp LP). O cálculo da taxa de incremento anual foi realizado utilizando *software* Microsoft Excel 2007. Os gráficos foram elaborados utilizando o *software* SPSS 25.0 (IBM Corp. Released 2017. IBM SPSS Statistics for Windows, Version 25.0. Armonk, NY: IBM Corp.).

Não houve necessidade de submissão ao Comitê de Ética em Pesquisa, uma vez que se trata de um estudo que utiliza dados secundários, sem identificação dos participantes. A Resolução n° 466 do Conselho Nacional de Saúde, de 12 de dezembro de 2012, foi atendida.

## RESULTADOS

Entre 2000 e 2016, ocorreram 485.015 óbitos específicos por lesões no trânsito no Brasil. Atribuídos aos *garbage codes* V87 a V89, foram 154.090 óbitos, redistribuídos entre os óbitos por lesões de trânsito com condição de vítima especificada. Para o V99 ocorreram 10.598 óbitos, dos quais foram acrescentados 10.534 à análise. Os indeterminados das causas externas (Y32 a Y34) foram 103.354 óbitos, dos quais foram redistribuídos 37.401 óbitos à análise, contabilizando 202.025 *garbage codes* redistribuídos para lesões no trânsito. Foram excluídos 38.717 óbitos cujo município de residência da vítima era ignorado. Dessa forma, foram analisados 648.322 óbitos. Em 206.338 (31,82%) desses a vítima estava na condição de pedestre, em 189.994 (29,30%) na condição de ocupante de motocicleta ou triciclo e em 188.569 (29,08%) na condição de ocupante de automóvel ou caminhonete.

Considerando também o período de 2000 a 2016, entre as capitais brasileiras, a maior taxa de mortalidade por ATT padronizada ocorreu em Boa Vista, RR (41,6/100 mil habitantes). A maior taxa de mortalidade específica na condição de pedestre foi observada no Macapá, AP (14,7/100 mil habitantes). Na condição de ocupante de motocicletas ou triciclos, a maior mortalidade também ocorreu em Boa Vista, RR (16,1/100 mil habitantes). A maior taxa específica para a condição de ocupantes de automóveis foi verificada em Palmas, TO (11,3/100 mil habitantes), conforme a Figura 1.

**Figura:** Mortalidade por acidentes de transporte terrestre geral e por condição da vítima segundo município de residência (capital e não capital). Brasil, 2000 e 2016.

Entre os municípios não capitais, aqueles que apresentaram maior taxa de mortalidade por ATT padronizada foram os do estado de Mato Grosso (35,6 óbitos/100 mil habitantes). Ainda dentre os municípios não capitais, a maior taxa específica de mortalidade na condição de pedestre ocorreu no Rio de Janeiro (10,2/100 mil habitantes). Na condição de ocupante de motocicleta ou triciclo, a maior taxa foi observada nos municípios do Piauí (17,2/100 mil habitantes). Na condição de ocupante de automóvel ou caminhonete, a maior taxa de mortalidade específica ocorreu nos municípios do Mato Grosso (12,1/100 mil habitantes). Esses dados podem ser observados na Figura 1.

A mortalidade por ATT (todas as condições da vítima) não apresentou tendência crescente em nenhuma das capitais no período em estudo. Já entre os municípios não capitais, a tendência foi crescente em 14 estados. Na Região Norte: Acre (TIA = 4,8 óbitos/100 mil habitantes), Amazonas (TIA = 4,7óbitos/100 mil habitantes), Pará (TIA = 4,1 óbitos/100 mil habitantes), Rondônia (TIA = 2,1 óbitos/100 mil habitantes) e Tocantins (TIA = 3,5 óbitos/100 mil habitantes). Na Região Nordeste, todos os estados, exceto o Rio Grande do Norte, apresentaram tendência crescente da mortalidade por ATT nos municípios não capitais. Nas regiões Sudeste e Sul não houve tendência crescente da mortalidade nas unidades geográficas em estudo. Na Região Centro-Oeste, houve tendência crescente apenas no Mato Grosso (TIA = 1,0 óbito/100 mil habitantes), como apresentado na Tabela 1.

**Tabela 1:** Taxa de incremento anual da mortalidade por ATT segundo município de residência da vítima. Brasil, 2000–2016.

Região	Capitais	Mortalidade por ATT				Não capitais*	Mortalidade por ATT			
		TIA	LI	LS	Tendência		TIA	LI	LS	Tendência
Norte	Rio Branco	-3,3	-6,1	-0,5	↓	AC	4,8	1,8	7,9	↑
	Macapá	-4,4	-6,6	-2,2	↓	AP	1,0	-1,1	3,2	–––
	Manaus	-1,7	-3,2	-0,2	↓	AM	4,7	2,5	6,9	↑
	Belém	-1,1	-2,3	0,1	–––	PA	4,1	3,2	4,9	↑
	Porto Velho	-2,5	-5,1	0,1	–––	RO	2,1	0,2	3,9	↑
	Boa Vista	-2,4	-4,2	-0,6	↓	RR	0,3	-1,8	2,4	–––
	Palmas	-1,4	-2,1	-0,6	↓	TO	3,5	2,4	4,6	↑
Nordeste	Maceió	-2,3	-3,6	-1,1	↓	AL	2,5	1,0	3,9	↑
	Salvador	0,4	-2,6	3,5	–––	BA	3,2	1,3	5,1	↑
	Fortaleza	-0,6	-2,1	0,9	–––	CE	3,4	1,9	4,9	↑
	São Luís	-0,2	-2,3	1,9	–––	MA	6,4	5,0	7,9	↑
	João Pessoa	-2,6	-3,5	-1,7	↓	PB	5,2	4,0	6,5	↑
	Recife	-1,0	-2,7	0,8	–––	PE	1,6	0,1	3,0	↑
	Teresina	0,7	-0,6	2,1	–––	PI	7,5	5,5	9,6	↑
	Natal	-3,3	-4,3	-2,1	↓	RN	0,7	-0,3	1,6	–––
	Aracaju	-3,3	-6,3	-0,2	↓	SE	1,7	0,0	3,4	↑
Sudeste	Vitória	-3,9	-5,4	-2,5	↓	ES	-0,6	-2,5	1,3	–––
	Belo Horizonte	-2,6	-5,1	0,1	–––	MG	1,5	-0,8	4,0	–––
	Rio de Janeiro	-1,8	-3,7	0,2	–––	RJ	-1,4	-3,0	0,3	–––
	São Paulo	-1,6	-3,8	0,7	–––	SP	-2,3	-3,3	-1,3	↓
Sul	Curitiba	-4,8	-6,6	-2,9	↓	PR	-0,3	-2,2	1,7	–––
	Porto Alegre	-3,4	-4,6	-2,3	↓	RS	-1,2	-2,4	0,0	↓
	Florianópolis	-4,2	-5,5	-2,8	↓	SC	-1,7	-3,8	0,4	–––
Centro-Oeste	Goiânia	-2,9	-4,2	-1,5	↓	GO	0,7	-0,2	1,6	–––
	Cuiabá	-0,1	-1,7	1,6	–––	MT	1,0	0,0	2,1	↑
	Campo Grande	-1,1	-2,9	0,6	–––	MS	1,3	-1,2	3,8	–––

* Municípios não capitais de cada estado.

ATT: acidentes de transporte terrestre; TIA: taxa de incremento anual; LI: limite inferior do intervalo de confiança de 95%; LS: limite superior do intervalo de confiança de 95%.

A taxa de mortalidade específica para pedestres não apresentou tendência crescente em nenhuma das unidades geográficas analisadas. Entretanto, houve estacionariedade das taxas em quatro capitais da Região Norte (Macapá, Porto Velho, Boa Vista e Palmas) e uma capital da Região Nordeste (Salvador). Em nove estados das regiões Norte e Nordeste houve estacionariedade das taxas em municípios não capitais. Nas regiões Centro-Oeste, Sudeste e Sul, a mortalidade específica na condição de pedestre foi decrescente em todas as análises (Tabela 2).

**Tabela 2:** Taxa de incremento anual da mortalidade de pedestres segundo município de residência da vítima. Brasil, 2000–2016.

Região	Capitais	Mortalidade de pedestres				Não capitais*	Mortalidade de pedestres			
		TIA	LI	LS	Tendência		TIA	LI	LS	Tendência
Norte	Rio Branco	-9,3	-12,9	-5,6	↓	AC	-1,6	-6,7	3,8	–––
	Macapá	-11,1	-22,4	1,8	–––	AP	-8,2	-13,8	-2,2	↓
	Manaus	-4,2	-8,1	-0,1	↓	AM	1,3	-1,5	4,2	–––
	Belém	-2,6	-4,4	-0,8	↓	PA	0,0	-1,8	1,9	–––
	Porto Velho	5,9	-3,4	16,0	–––	RO	-2,2	-3,5	-1,0	↓
	Boa Vista	-3,8	-9,8	2,7	–––	RR	2,2	-2,1	6,7	–––
	Palmas	-2,8	-7,6	2,3	–––	TO	-0,8	-3,0	1,5	–––
Nordeste	Maceió	-7,7	-10,6	-4,6	↓	AL	-4,1	-6,2	-2,0	↓
	Salvador	-1,7	-5,7	2,5	–––	BA	-1,2	-3,4	1,1	–––
	Fortaleza	-3,2	-5,7	-0,6	↓	CE	-1,6	-4,3	1,2	–––
	São Luís	-5,1	-7,7	-2,3	↓	MA	-0,4	-3,0	2,2	–––
	João Pessoa	-7,1	-8,9	-5,2	↓	PB	-5,8	-8,1	-3,5	↓
	Recife	-4,4	-6,2	-2,6	↓	PE	-3,7	-4,8	-2,6	↓
	Teresina	-5,7	-6,4	-5,0	↓	PI	-1,9	-4,1	0,4	–––
	Natal	-6,4	-11,2	-1,3	↓	RN	-5,8	-7,4	-4,2	↓
	Aracaju	-5,3	-8,4	-2,0	↓	SE	-4,4	-6,7	-2,0	↓
Sudeste	Vitória	-5,7	-7,4	-3,9	↓	ES	-5,6	-7,2	-4,0	↓
	Belo Horizonte	-6,3	-9,5	-3,1	↓	MG	-3,4	-5,3	-1,4	↓
	Rio de Janeiro	-3,8	-5,6	-2,0	↓	RJ	-5,4	-7,2	-3,7	↓
	São Paulo	-5,9	-8,0	-3,9	↓	SP	-7,8	-9,4	-6,1	↓
Sul	Curitiba	-7,4	-8,8	-5,9	↓	PR	-4,7	-6,4	-3,0	↓
	Porto Alegre	-7,2	-8,9	-5,4	↓	RS	-6,9	-7,8	-6,0	↓
	Florianópolis	-8,1	-10,4	-5,8	↓	SC	-6,7	-8,3	-5,2	↓
Centro-Oeste	Goiânia	-5,6	-7,9	-3,3	↓	GO	-5,1	-7,3	-3,0	↓
	Cuiabá	-5,7	-9,0	-2,2	↓	MT	-4,4	-5,7	-3,1	↓
	Campo Grande	-9,6	-11,6	-7,6	↓	MS	-5,8	-6,9	-4,6	↓

* Municípios não capitais de cada estado.

ATT: acidentes de transporte terrestre; TIA: taxa de incremento anual; LI: limite inferior do intervalo de confiança de 95%; LS: limite superior do intervalo de confiança de 95%.

A taxa de mortalidade específica para ocupantes de motocicletas ou triciclos não apresentou tendência decrescente em nenhuma das unidades geográficas em estudo. Houve tendência estacionária das taxas em 10 capitais. Na Região Norte: Rio Branco, Porto Velho e Boa Vista. Na Região Nordeste: Aracaju. Na Região Sudeste: Vitória e Belo Horizonte. Na Região Centro-Oeste: Goiânia. Na Região Sul todas as capitais apresentaram tendência estacionária. Considerando os municípios não capitais, apenas quatro estados apresentaram tendência estacionária das taxas de mortalidade para ocupantes de motocicletas ou triciclos: Roraima, Paraná, Rio Grande do Sul e Santa Catarina (Tabela 3).

**Tabela 3:** Taxa de incremento anual da mortalidade de ocupantes de motocicletas/triciclos segundo município de residência da vítima. Brasil, 2000–2016.

Região	Capitais	Mortalidade de ocupantes de motocicletas e triciclos				Não capitais*	Mortalidade de ocupantes de motocicletas e triciclos			
		TIA	LI	LS	Tendência		TIA**	LI	LS	Tendência
Norte	Rio Branco	3,1	-9,8	17,8	–––	AC	12,1	6,6	17,8	↑
	Macapá	14,0	7,5	20,9	↑	AP	16,6	10,5	23,1	↑
	Manaus	4,7	2,1	7,4	↑	AM	8,5	5,3	11,8	↑
	Belém	14,9	10,6	19,3	↑	PA	11,7	10,4	12,9	↑
	Porto Velho	-3,2	-9,1	3,0	–––	RO	6,8	3,2	10,6	↑
	Boa Vista	0,6	-5,3	6,8	–––	RR	2,1	-3,4	7,8	–––
	Palmas	2,7	0,4	5,0	↑	TO	9,1	7,1	11,2	↑
Nordeste	Maceió	13,9	7,4	20,9	↑	AL	14,5	9,7	19,6	↑
	Salvador	17,3	10,6	24,5	↑	BA	12,8	11,1	14,7	↑
	Fortaleza	4,5	0,2	9,1	↑	CE	7,8	5,3	10,4	↑
	São Luís	9,8	6,3	13,4	↑	MA	15,2	12,8	17,6	↑
	João Pessoa	12,9	7,4	18,7	↑	PB	15,0	8,3	22,1	↑
	Recife	8,8	5,1	12,7	↑	PE	9,8	7,2	12,4	↑
	Teresina	7,9	5,7	10,1	↑	PI	11,1	8,2	14,0	↑
	Natal	4,9	0,5	9,6	↑	RN	7,6	5,6	9,6	↑
	Aracaju	3,5	-3,9	11,5	–––	SE	11,7	6,4	17,3	↑
Sudeste	Vitória	2,5	-1,7	6,9	–––	ES	6,9	2,5	11,4	↑
	Belo Horizonte	2,3	-1,7	6,4	–––	MG	8,4	3,4	13,7	↑
	Rio de Janeiro	9,8	0,5	20,0	↑	RJ	6,3	2,0	10,9	↑
	São Paulo	8,6	3,4	14,0	↑	SP	5,0	0,8	9,3	↑
Sul	Curitiba	3,1	-2,1	8,6	–––	PR	4,6	-0,2	9,6	–––
	Porto Alegre	2,9	-0,3	6,3	–––	RS	3,4	-1,5	8,6	–––
	Florianópolis	2,8	-0,7	6,5	–––	SC	3,2	-1,8	8,5	–––
Centro-Oeste	Goiânia	0,1	-2,2	2,5	–––	GO	6,0	4,0	8,0	↑
	Cuiabá	8,7	6,3	11,1	↑	MT	6,3	2,8	9,9	↑
	Campo Grande	5,1	2,5	7,8	↑	MS	7,5	2,6	12,7	↑

* Municípios não capitais de cada estado.

ATT: acidentes de transporte terrestre; TIA: taxa de incremento anual; LI: limite inferior do intervalo de confiança de 95%; LS: limite superior do intervalo de confiança de 95%.

Das capitais, a única que apresentou tendência crescente da mortalidade específica de ocupantes de automóveis ou caminhonetes foi Teresina (TIA = 9,5 óbitos/100 mil habitantes). Dentre os municípios não capitais, nove apresentaram tendência crescente. Na Região Norte: Amazonas (TIA = 5,8 óbitos/100 mil habitantes), Pará (TIA = 1,5 óbitos/100 mil habitantes) e Tocantins (TIA = 2,4 óbitos/100 mil habitantes). Na Região Nordeste: Ceará (TIA = 2,9 óbitos/100 mil habitantes), Paraíba (TIA = 5,1 óbitos/100 mil habitantes) e Piauí (TIA = 14,0 óbitos/100 mil habitantes). Na Região Sudeste: Minas Gerais (TIA = 2,6 óbitos/100 mil habitantes). Na Região Sul: Rio Grande do Sul (TIA = 2,1 óbitos/100 mil habitantes). Na Região Centro-Oeste: Mato Grosso do Sul (TIA = 4,6 óbitos/100 mil habitantes). Houve decréscimo da mortalidade de ocupantes de automóveis ou caminhonetes em seis capitais. No Nordeste: Natal (TIA = −4,3 óbitos/100 mil habitantes) e Aracaju (TIA = −6,7 óbitos/100 mil habitantes). Na Região Sul: Curitiba (TIA = −5,4 óbitos/100 mil habitantes) e Florianópolis (TIA = −5,5 óbitos/100 mil habitantes). Na Região Centro-Oeste: Goiânia (TIA = −3,1 óbitos/100 mil habitantes) e Cuiabá (TIA = −1,9 óbitos/100 mil habitantes). Entre os municípios não capitais, houve decréscimo da taxa apenas no Rio Grande do Norte (TIA = −4,3 óbitos/100 mil habitantes) e Sergipe (TIA = −3,7 óbitos/100 mil habitantes), ambos na Região Nordeste. As demais unidades geográficas em análise apresentaram tendência estacionária das taxas de mortalidade específicas de ocupantes de automóveis ou caminhonetes no período (Tabela 4).

**Tabela 4:** Taxa de incremento anual da mortalidade de ocupantes de automóveis ou caminhonetes segundo município de residência da vítima. Brasil, 2000–2016.

Região	Capitais	Mortalidade de ocupantes de automóveis e caminhonetes				Não capitais*	Mortalidade de ocupantes de automóveis e caminhonetes			
		TIA	LI	LS	Tendência		TIA	LI	LS	Tendência
Norte	Rio Branco	-1,0	-11,4	10,7	–––	AC	1,8	-5,1	9,3	–––
	Macapá	1,9	-7,5	12,1	–––	AP	3,9	-3,1	11,5	–––
	Manaus	-1,4	-4,2	1,4	–––	AM	5,8	1,6	10,2	↑
	Belém	-2,6	-8,5	3,6	–––	PA	1,5	0,5	2,5	↑
	Porto Velho	-0,4	-11,3	11,8	–––	RO	-0,4	-4,0	3,3	–––
	Boa Vista	-3,9	-7,9	0,2	–––	RR	-1,4	-8,1	5,9	–––
	Palmas	-2,1	-6,0	2,0	–––	TO	2,4	0,8	4,1	↑
Nordeste	Maceió	-1,1	-5,1	3,1	–––	AL	-1,1	-5,8	3,8	–––
	Salvador	-4,0	-9,4	1,8	–––	BA	1,0	-1,6	3,7	–––
	Fortaleza	-0,5	-3,2	2,3	–––	CE	2,9	1,2	4,7	↑
	São Luís	-0,8	-6,7	5,5	–––	MA	1,1	-1,2	3,5	–––
	João Pessoa	-4,7	-9,9	0,9	–––	PB	5,1	1,8	8,5	↑
	Recife	0,6	-4,4	5,9	–––	PE	0,4	-0,7	1,4	–––
	Teresina	9,5	5,8	13,2	↑	PI	14,0	8,7	19,6	↑
	Natal	-6,7	-10,6	-2,6	↓	RN	-4,3	-5,3	-3,3	↓
	Aracaju	-6,7	-12,0	-1,0	↓	SE	-3,7	-6,6	-0,7	↓
Sudeste	Vitória	-9,4	-19,5	1,9	–––	ES	-1,3	-3,8	1,2	–––
	Belo Horizonte	0,9	-1,9	3,8	–––	MG	2,6	0,3	4,9	↑
	Rio de Janeiro	-1,2	-6,1	3,9	–––	RJ	2,2	-1,3	5,9	–––
	São Paulo	-0,1	-2,1	1,9	–––	SP	0,0	-2,5	2,6	–––
Sul	Curitiba	-5,4	-7,2	-3,6	↓	PR	1,1	-0,5	2,7	–––
	Porto Alegre	-1,0	-3,2	1,3	–––	RS	2,1	0,9	3,2	↑
	Florianópolis	-5,5	-7,1	-3,8	↓	SC	-0,6	-1,7	0,5	–––
Centro-Oeste	Goiânia	-3,1	-5,7	-0,3	↓	GO	0,5	-2,0	3,1	–––
	Cuiabá	-1,9	-3,8	0,0	↓	MT	-0,6	-2,7	1,6	–––
	Campo Grande	0,1	-3,5	3,9	–––	MS	4,6	1,2	8,0	↑

* Municípios não capitais de cada estado.

ATT: acidentes de transporte terrestre; TIA: taxa de incremento anual; LI: limite inferior do intervalo de confiança de 95%; LS: limite superior do intervalo de confiança de 95%.

## DISCUSSÃO

No período de 2000 a 2016, os municípios não capitais apresentaram tendências desfavoráveis das taxas padronizadas de mortalidade por ATT na comparação com as capitais de seus respectivos estados. Essa discrepância foi mais acentuada nas macrorregiões mais pobres do país (Norte e Nordeste). Os acidentes envolvendo motocicletas foram a categoria em que foi observada tendência crescente mais acentuada, o que reflete a interiorização do uso deste tipo de veículo no país.

Houve preponderância de tendência estacionária (observada em 12 capitais e 10 aglomerados de não capitais) ou de redução (observada em 14 capitais e 2 aglomerados de não capitais) das taxas de mortalidade por ATT no Brasil no período em estudo. Essa tendência pode ter sido causada por melhorias na infraestrutura viária, redução da velocidade média (tanto pelo aumento do fluxo de veículos quanto pela implementação de medidas de controle e fiscalização da velocidade), aumento do uso de equipamentos de segurança (capacete, cinto de segurança, equipamento de controle de crianças etc.), diminuição de fatores de risco como “beber e dirigir” e também pela melhoria no atendimento pré-hospitalar e hospitalar às vítimas^13^. A redução da magnitude da mortalidade de pedestres segue uma tendência mundial e está relacionada a esses mesmos fatores^2^^,^^22^. A redução pode ser explicada, também, pela expansão das campanhas de educação no trânsito e ênfase no uso da faixa de pedestres^23^.

De acordo com o Anuário Estatístico do Seguro de Danos Pessoais Causados por Veículos Automotores de Vias Terrestres, a partir de 2012 houve uma redução no número de indenizações pagas por óbitos em acidentes de trânsito em todo o Brasil. Souza et al.^24^ relataram um aumento da taxa padronizada de mortalidade por acidentes de motocicleta e atropelamentos no período de 1980 a 2003 no Brasil. Nesse mesmo período, os autores observaram redução da mortalidade por acidentes envolvendo outros meios de transporte terrestre. Morais Neto et al.^22^ relataram que a taxa de mortalidade por ATT no Brasil variou de 18,2 por 100.000 habitantes em 2000 para 22,54 por 100.000 habitantes em 2010, representando um aumento de 22,54%. Scolari et al.^25^ observaram redução na taxa de mortalidade por ATT no Brasil entre os anos de 2006 e 2008, tanto considerando como denominador o número de habitantes quanto considerando a frota de veículos.

O aumento marcante da mortalidade de motociclistas, principalmente nas regiões Norte, Nordeste e Centro-Oeste, pode estar relacionado ao rápido crescimento da frota de veículos. Embora tenha ocorrido em todo o país, ele foi mais marcante nas três regiões citadas^7^^,^^26^^–^^29^. Principalmente em municípios do interior, a motocicleta vem sendo utilizada em substituição a veículos não motorizados, como a bicicleta e animais^13^.

À vulnerabilidade da exposição corporal dos motociclistas, soma-se o comportamento de risco adotado por eles, o que aumenta consideravelmente os riscos de lesões e mortes. A Pesquisa Nacional de Saúde de 2013 mostrou que, nas regiões Norte, Nordeste e Centro-Oeste, o uso de capacete é menos frequente que nas demais regiões. O uso do capacete reduz o risco de morte em 40% e o risco de lesão em 70%. Além disso, as regiões Norte e Nordeste apresentam o menor percentual de municípios inseridos no Sistema Nacional de Trânsito. Como consequência, possuem menor capacidade de fiscalização e investimento na sinalização e manutenção da infraestrutura viária^30^.

A desigualdade entre as tendências de mortalidade por ATT em capitais e municípios não capitais de cada estado entre 2000 e 2016 pode ser explicada pelo aumento desigual da frota e pela capacidade desigual de implementação de medidas regulatórias no trânsito. A facilidade de aquisição de veículos motorizados, em especial motocicletas, foi favorecida pelos benefícios tributários implementados pelo governo federal tanto no que diz respeito à sua produção quanto ao seu consumo^25^. Adicionalmente, há ineficácia ou mesmo inexistência de transporte público em muitos municípios, e as políticas de mobilidade urbana privilegiam o transporte individual e privado em detrimento do transporte coletivo público^26^. Esses fatores, associados ao crescimento econômico, fizeram com que a frota de veículos motorizados no Brasil saltasse de 29.722.950 para 93.867.016 entre 2000 e 2016. As motocicletas, que no início desse período correspondiam a 11,90% do total da frota nacional, passaram a representar 22,31% em 2016, permanecendo em 22,22% em 2018^27^.

Nas últimas décadas, houve um crescente reforço da legislação relacionada à prevenção dos fatores de risco para mortes e lesões no trânsito. O Código de Trânsito Brasileiro foi instituído em 1998, procurando circunscrever os principais fatores de risco comportamentais para ATT (velocidade, direção após consumo de bebida alcoólica e não uso do capacete, do cinto de segurança e do dispositivo para transporte de crianças)^31^^,^^32^. No entanto, sua progressiva implementação nos anos seguintes ocorreu de modo desigual entre as capitais e municípios não capitais, assim como entre as macrorregiões, de acordo com suas condições diferenciais para implantar e fiscalizar o sistema de trânsito^33^. Desse modo, seus possíveis efeitos favoráveis para a redução da mortalidade por ATT também se deram de modo desigual e os resultados aqui descritos são compatíveis com essa hipótese^34^.

A capacidade de fiscalização dos órgãos do SNT, por exemplo, ainda é insuficiente nas três esferas de gestão do país^7^. Na esfera municipal, apenas 28,22% dos municípios não capitais integram o SNT. Enquanto isso, 100% das capitais aderiram à municipalização do trânsito^11^. A comparação entre as taxas de mortalidade por ATT nos anos de 2000 e 2015 mostrou menor crescimento da taxa de mortalidade entre os municípios com trânsito integrado ao SNT (variação percentual igual a 8,5%) em comparação com os não integrados (29,1%)^13^.

Em 2001, foi criada a Política Nacional de Redução da Morbimortalidade por Acidentes e Violências (Portaria MS/GM n° 737 de 16 de maio de 2001), estabelecendo a necessidade de intervenções que promovessem a adoção de comportamentos e de ambientes seguros e saudáveis. Em 2004, foi publicada a Política Nacional de Trânsito (Resolução n° 166, de 15 de setembro de 2004), que inseriu o trânsito no plano global de governo e apresentou objetivos, diretrizes e metas para o seu aprimoramento no Brasil. Em 2010, foi criado o Projeto Vida no Trânsito, coordenado pelo Ministério da Saúde. O projeto pressupunha a intervenção prioritária em dois fatores de risco para os acidentes de trânsito: velocidade excessiva e inadequada e condução de veículos após consumo de bebida alcoólica. O projeto se transformou em Programa Vida no Trânsito e, em 2013, foi expandido^6^. Além disso, em 2011, o Departamento Nacional de Trânsito implantou o Pacto Nacional pela Redução de Acidentes de Trânsito, com participação do Ministério da Saúde e o Ministério das Cidades^35^. Todas estas medidas provavelmente contribuíram para o declínio dos indicadores de mortalidade por ATT, principalmente nas regiões mais ricas do país e nas capitais, que contam com mais recursos e possivelmente conseguiram implantá-los de modo mais rápido e extenso em seus territórios^36^. Isso poderia explicar, também, as diferenças de mortalidade entre os meios de transporte, entre as cidades capitais e não capitais e entre as macrorregiões. No caso da mortalidade de ocupantes de motocicletas, o aumento marcante da frota se sobrepôs às políticas públicas acima citadas^37^. Desta forma, a mortalidade por essa causa foi crescente na maior parte das unidades aqui analisadas.

A ausência de informações sobre diferenciais de implantação do Código de Trânsito Brasileiro entre as cidades e entre as macrorregiões é uma limitação deste estudo. Também pode ser considerada uma limitação do estudo o fato de haver diferenças de qualidade na identificação das causas de morte^38^. No Sistema de Informações sobre Mortalidade do Brasil, há a possibilidade de sub-registro das causas de óbito, principalmente nos municípios pequenos e nas macrorregiões mais pobres do país, o que pode afetar parcialmente os resultados de modo não controlado.

Os resultados deste artigo sugerem que as políticas públicas de prevenção de acidentes não têm sido tão eficazes quanto as políticas de mercado para o aumento da venda de veículos. A fiscalização permanente do cumprimento da legislação de trânsito e os investimentos em engenharia de tráfego são ações fundamentais para reverter esse quadro. Devem ser implementadas, também, ações relacionadas à mobilidade urbana por meio da melhoria da qualidade e frequência do transporte público coletivo e construção de ciclofaixas adequadas. Essas medidas precisam ser implementadas de maneira uniforme entre os municípios e regiões, de forma a reduzir as desigualdades que foram aqui observadas.
